# Bioinspired Jellyfish Microparticles from Microfluidics

**DOI:** 10.34133/research.0034

**Published:** 2023-01-16

**Authors:** Chaoyu Yang, Yunru Yu, Yuanjin Zhao, Luoran Shang

**Affiliations:** ^1^ Department of Clinical Laboratory, Nanjing Drum Tower Hospital, School of Biological Science and Medical Engineering, Southeast University, Nanjing 210096, China.; ^2^ Oujiang Laboratory (Zhejiang Lab for Regenerative Medicine, Vision and Brain Health), Wenzhou Institute, University of Chinese Academy of Sciences, Wenzhou, Zhejiang 325001, China.; ^3^ Shanghai Xuhui Central Hospital, Zhongshan-Xuhui Hospital, and the Shanghai Key Laboratory of Medical Epigenetics, the International Co-laboratory of Medical Epigenetics and Metabolism (Ministry of Science and Technology), Institutes of Biomedical Sciences, Fudan University, Shanghai, China.

## Abstract

Nonspherical particles have attracted increasing interest because of their shape anisotropy. However, the current methods to prepare anisotropic particles suffer from complex generation processes and limited shape diversity. Here, we develop a piezoelectric microfluidic system to generate complex flow configurations and fabricate jellyfish-like microparticles. In this delicate system, the piezoelectric vibration could evolve a jellyfish-like flow configuration in the microchannel and the in situ photopolymerization could instantly capture the flow architecture. The sizes and morphologies of the particles are precisely controlled by tuning the piezoelectric and microfluidic parameters. Furthermore, multi-compartmental microparticles with a dual-layer structure are achieved by modifying the injecting channel geometry. Moreover, such unique a shape endows the particles with flexible motion ability especially when stimuli-responsive materials are incorporated. On the basis of that, we demonstrate the capability of the jellyfish-like microparticles in highly efficient adsorption of organic pollutants under external control. Thus, it is believed that such jellyfish-like microparticles are highly versatile in potential applications and the piezoelectric-integrated microfluidic strategy could open an avenue for the creation of such anisotropic particles.

## Introduction

Microparticles have demonstrated substantial application values in a large number of areas [1–4]. Commonly, the functions and properties of the particles are closely interrelated with their structures and morphologies [5–7]. Nonspherical particles possess unique features originating from their shape anisotropy such as optical properties and motion characteristics, which bring about fascinating potential applications including sensing, catalysis, and microactuators [8–13]. Currently, numerous engineering techniques are developed to prepare nonspherical microparticles such as stretching methods [14], lithography, micromolding, etc. [15–18]. The resultant particles have demonstrated their values as photonic materials, drug release vehicles, micromotors, and catalysts [19–23]. Although with many successes, a large portion of the above methods rely on complex equipment and sophisticated generation processes, and it is difficult to fabricate large quantities of anisotropic particles in a fast and efficient manner. Moreover, the morphological diversity of the resultant particles remains to be explored. Thus, novel preparation strategies for the continuous preparation of anisotropic particles are still anticipated.

**Fig. 1.**
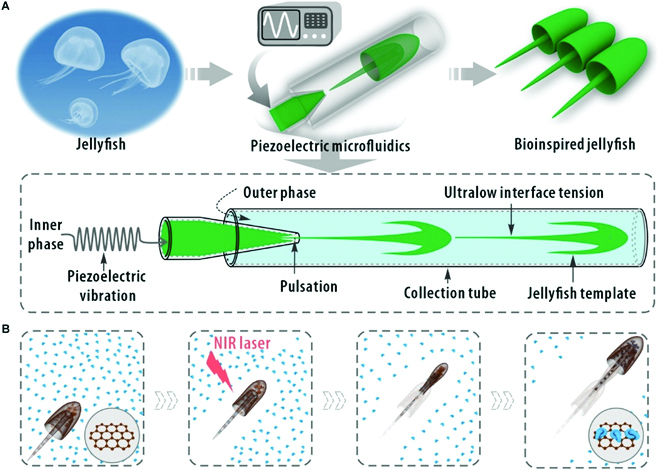
Schematic illustrations of the generation and adsorption process of the jellyfish particles. (A) Schematic diagram of jellyfish, the piezoelectric microfluidic setup, and the bioinspired jellyfish microparticles. The bottom panel demonstrates the preparation process of the bioinspired jellyfish. (B) Schematic diagram of the movement of a stimuli-responsive microparticle under NIR laser irradiation and the adsorption process for water contaminants.

Here, we presented novel jellyfish-inspired microparticles from microfluidics, as schemed in Fig. 1. Jellyfish are among the most representative of living organisms showing extremely irregular and exquisite shapes [24,25]. The unique umbrella-like shape of jellyfish facilitates their motion by radially expanding/contracting their body and, thus, pushing water behind [26,27]. This complex morphology is difficult to reproduce by conventional molding or templating methods. Alternatively, microfluidic is an emerging technique that masters precise control of fluid flow in constrained channels [28–30]. Benefiting from this, microfluidics has emerged as a promising method for the generation of various particles [31–36]. However, because the particles are mainly derived from fluid templates formed by oil–water emulsification, typically droplets, their shapes are generally spherical or slightly deviated from spherical because of the action of interfacial tension [37–42]. Thus, it is conceived that by introducing new fluid regimes through external forces, jellyfish-like particles could be prepared continuously in microfluidic systems.

In this paper, we employed a pulse-generating microfluidic system to generate complex flow configurations and fabricate jellyfish-like microparticles. By combining piezoelectric vibration with co-flow microfluidics, a photocurable fluid thread with periodic reentrance cavities was generated, which then evolved to jellyfish-like liquid templates and eventually solid particles through in situ ultraviolet (UV) polymerization. By adjusting the piezoelectric parameters, the morphology and size of the particles could be easily controlled. Furthermore, by incorporating graphene oxide/N-isopropylacrylamide (GO/NIPAM), the microparticles exhibited stimuli-responsive shrinkage behavior under near-infrared (NIR) irradiation. Owing to the anisotropic particle shape, such deformation could provide a propulsive thrust to propel the particle, in a way mimicking the jellyfish. Moreover, by further incorporating magnetic nanoparticles, the direction of movement of the particles could be controlled by a magnet. On the basis of that, we demonstrated the capability of the jellyfish-like microparticles in highly efficient adsorption of organic pollutants under external control. These results indicated the superiority of the pulse-generating microfluidic system in preparing highly anisotropic particles with implications for various applications.

## Results

**Fig. 2.**
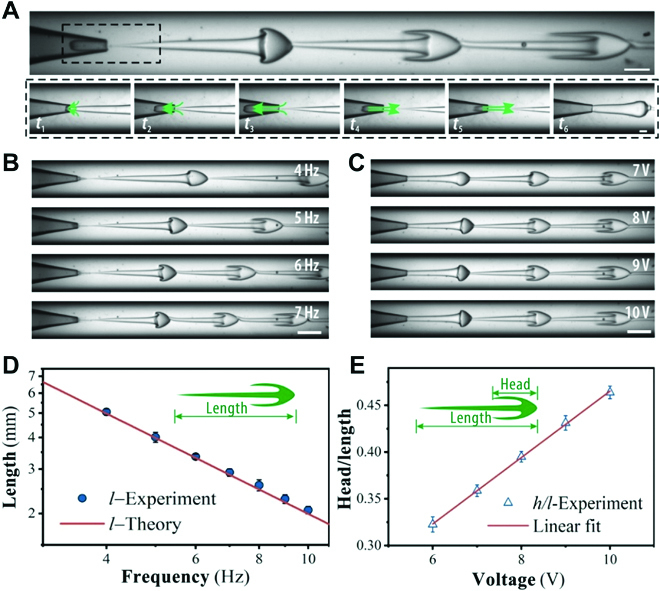
Generation dynamics and morphology control of the jellyfish-like ligament template in microfluidics. (A) High-speed real-time images of the generation of jellyfish ligament template in the microfluidic collection tube. The bottom panels show an entire process of fluid retraction and advancement. (B and C) High-speed real-time images of the dynamic behaviors of the jellyfish ligament template under different (B) piezoelectric frequencies and (C) actuation amplitudes. (D) Plot of the length of the jellyfish-like ligament template as a function of the piezoelectric frequency. (E) Plot of the ratio of the head to the length of the ligament template as a function of the piezoelectric voltage amplitude at the position of the third jellyfish in (C). The scale bars are 500 μm in the upper panel of (A), 250 μm in the bottom panels of (A), and 1000 μm in (B) and (C).

In a typical experiment, we assembled a co-flow microfluidic device by coaxially inserting a tapered injection capillary into an outer collection capillary (Fig. S1). An inner aqueous pre-gel solution (polyethylene glycol-diacrylate, PEGDA) and the outer fluid of deionized water were simultaneously driven to the injection and collection capillary, respectively, and flowed in the same direction to form a steady jet formed downstream in the collection capillary. A piezoelectric stack (PSt150/7/20VS12, CoreMorrow) was employed to exert oscillation pulsations to the inner phase by a PTFE (polytetrafluoroethylene) film, and the inner phase fluid was modulated as desired (Fig. S2). When the amplitude of pulsation exceeded a certain value, the fluctuation caused the inner jet to periodically retract and go forward and then formed ligaments with a varicose head and thin-thread tail (Fig. 2A). Because of the large inertial force relative to the interfacial tension of the 2 aqueous phases [43], the ligaments continued to deform into a jellyfish-like shape in accordance with Poiseuille advection (Movie S1 and Note S1). Two key elements of preparing such unique particles are the low interfacial tension of the liquid–liquid system for the ease of deformation and the rapid solidification of the liquid for keeping the configuration. Then, the effects of the piezoelectric and the microfluidic parameters on the jellyfish-like ligament were systematically investigated in detail (Fig. 2B and C). It was found that the length *l* and head ratio *h/l* could be well controlled by changing the piezoelectric frequency and voltage, respectively, as plotted in Fig. 2D and E. Meanwhile, the inner flow rate would significantly influence the width of the ligament template (Fig. S3). These results demonstrated that, by simply programming the piezoelectric signals, the jellyfish-like ligament templates with controllable morphologies could be obtained on demand.

**Fig. 3.**
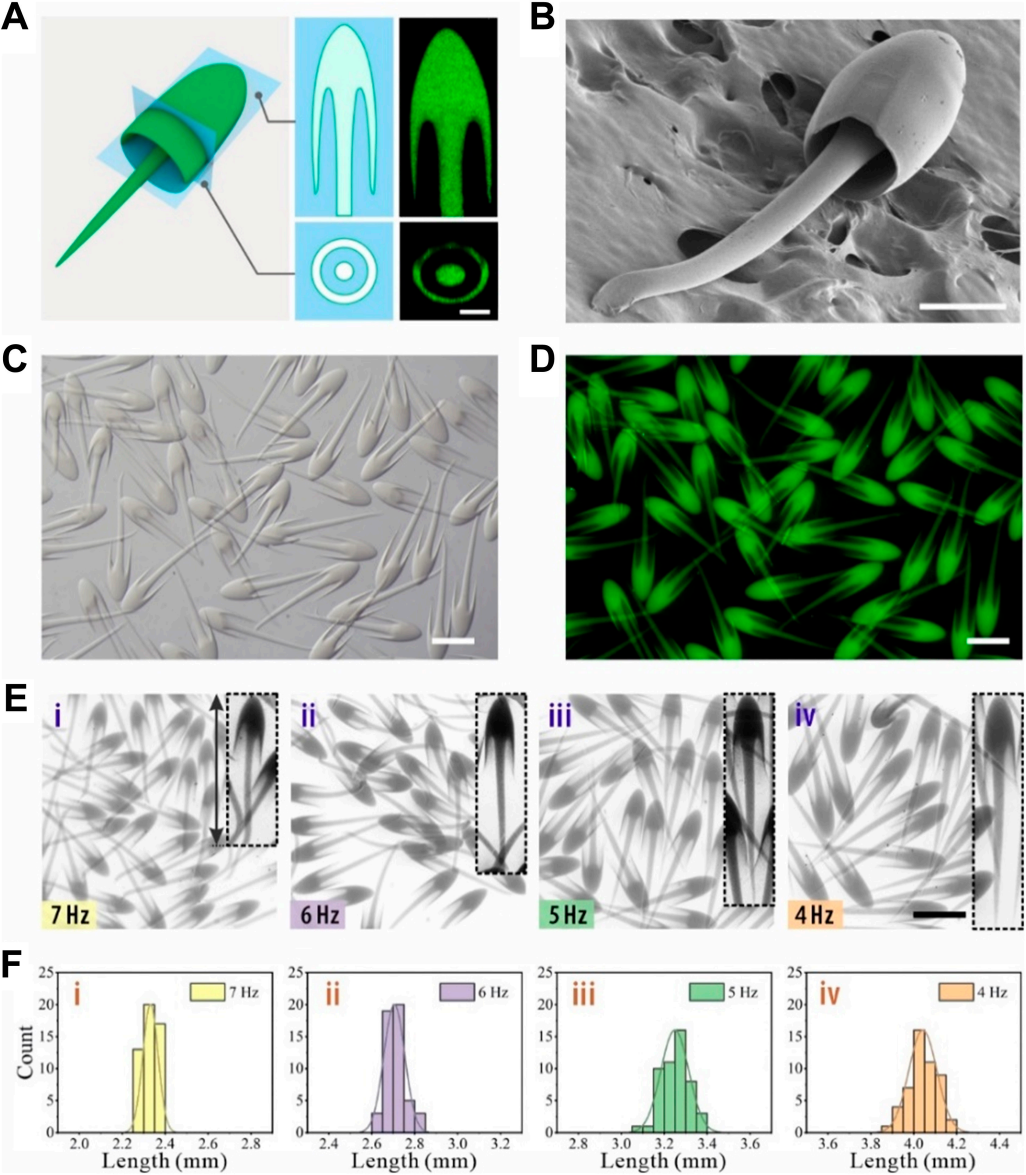
Formation of the single-layered jellyfish-like particles in microfluidics. (A) Schematic illustrations and cross-sectional confocal laser scanning microscope (CLSM) images of the resultant particle. (B) SEM image of one jellyfish particle. (C and D) Microscopy images of a batch of bioinspired particles with fluorescent polystyrene nanoparticles (E) in bright field and (D) fluorescent field. (E) Jellyfish microparticles produced under different piezoelectric frequencies. (F) The corresponding length distributions of the particles. The scale bars are 100 μm in (A), 200 μm in (B), and 1000 μm in (C) to (E).

After rapid solidification by UV irradiation, the jellyfish-like ligament templates were polymerized into solid particles with the same shape. The resultant particles possessed an umbrella-like head with an internal cavity and a trailing tentacle and showed component homogeneity (Fig. 3A). The morphology was further confirmed by scanning electron microscopy (SEM), as depicted in Fig. 3B. Because the morphology of the ligament templates evolved from bullet to jellyfish at the microfluidic channel, by shinning UV at different positions of the collection tube, the resultant particles could preserve the corresponding morphologies (Fig. S4). Benefiting from the continuous fluid flow in microfluidics, this method enabled consecutive generation of jellyfish-like particles with uniform size and morphology, as shown in Fig. 3C and D. Moreover, the jellyfish particle length could be finely tuned by altering the piezoelectric frequency, as shown in Fig. 3E. The length distribution of the jellyfish particles demonstrated monodispersity, with a coefficient of variation of <5% (the SD divided by the mean value), as plotted in Fig. 3F.

**Fig. 4.**
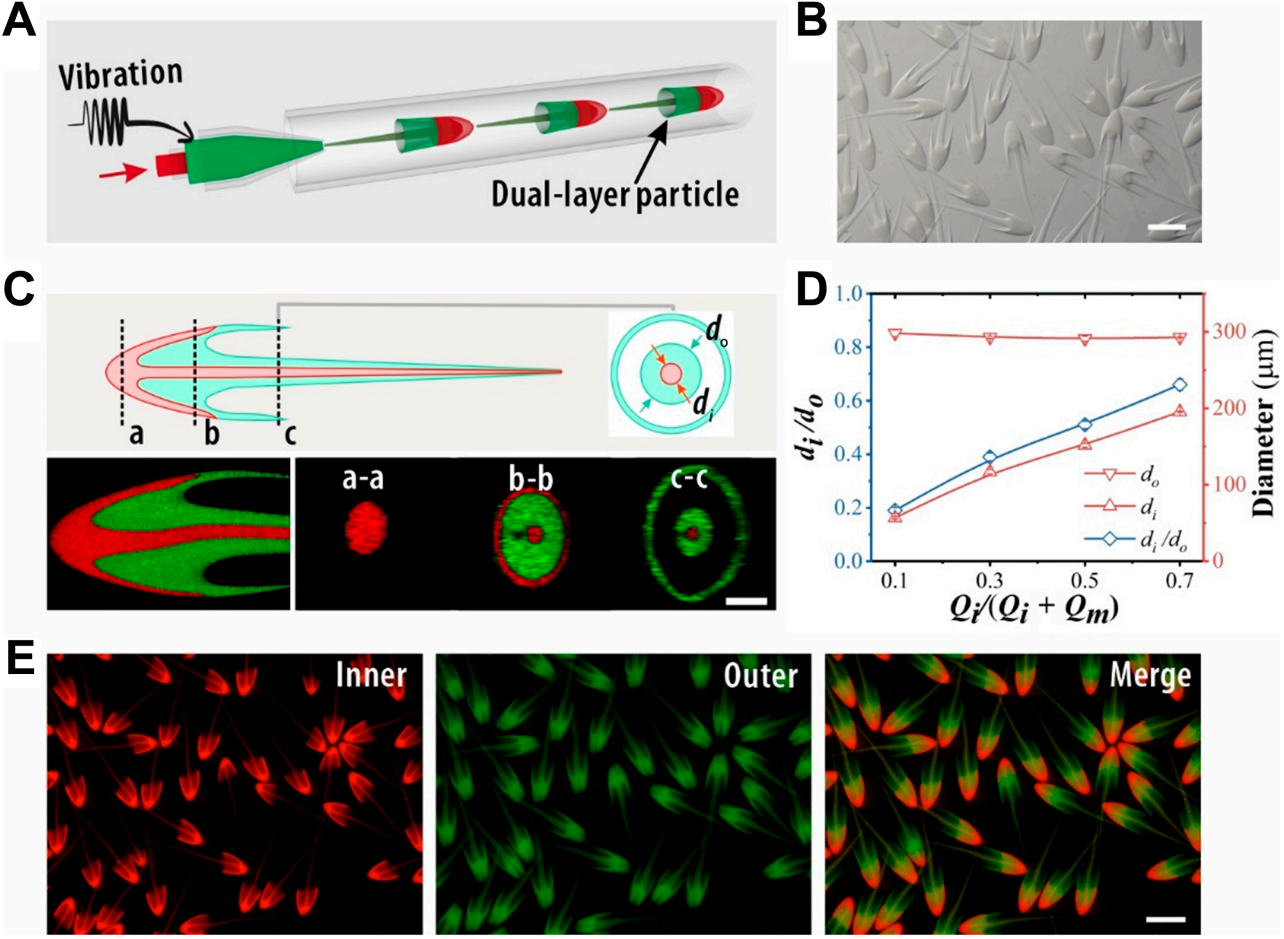
Formation of the dual-layered jellyfish-like particles in microfluidics. (A) Schematic illustration of the 2-stage nested capillary microfluidic device for the generation of dual-layered jellyfish-like microparticles. (B) Microscopy images of a batch of bioinspired dual-layered particles. (C) Schematic illustrations of the dual-layered particles with longitudinal and cross-sectional CLSM images. (D) The ratio of the internal thread diameter *d_i_* to the outer thread diameter *d_o_* as a function of *φ*. (E) Fluorescent images of a batch of dual-layered particles with red and green fluorescent polystyrene nanoparticles added to the inner and outer layer, respectively. The scale bars are 1000 μm in (B) and (E) and 200 μm in (C).

Intriguingly, by taking advantage of the flexibility in fluidic manipulation of microfluidics, we prepared dual-layered jellyfish-like microparticles. Specifically, a capillary microfluidic device was constructed, comprising a 2-stage nested channel design (Fig. S5), and the piezoelectric vibration was induced to the middle phase (Fig. 4A). The 2 inner fluids were pumped to the microfluidic channel and pulsed periodically, forming a dual-layered stream, which eventually resulted in the formation of dual-layered jellyfish-like microparticles (Fig. 4B). We demonstrated the dual-layered structure of the particles in both the head and the tail through a confocal laser scanning microscope (CLSM) (Fig. 4C and Movie S2). Furthermore, by tuning the microfluidic parameters (the innermost phase flow rate *Q_i_* and the middle phase flow rate *Q_m_*), the proportion of the 2 components could be controlled by *φ = Q_i_/*(*Q_i_ + Q_m_*), as shown in Fig. S6. To keep the morphology and size of the dual-layered particles consistent, the dispersed flow rate *Q_i_ + Q_m_* was fixed, and it was found that the increase of *φ* would increase the inner layer thickness, as plotted in Fig. 4D. Similar to that of the simple jellyfish microparticles, the dual-layered jellyfish-like particles could also be continuously fabricated with uniform size and morphology, as shown in Fig. 4E. Such dual-layered structure of the particles could facilitate the addition of functional ingredients as needed, thus supporting various functionalities.

**Fig. 5.**
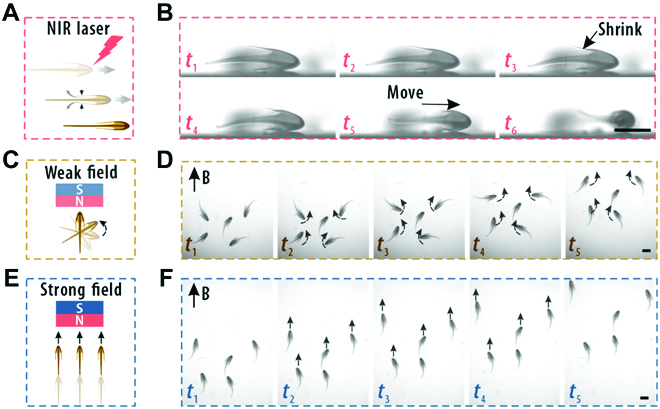
Stimuli-triggered motion of the dual-layered particles. (A and B) Schematic illustration and real-time images showing the movement of a single dual-layered jellyfish microparticle under NIR laser irradiation. (C and D) Schematic illustration and real-time images showing the reorientation of the magnetic-responsive dual-layered jellyfish microparticles under a weak magnetic field. (E and F) Schematic illustration and real-time images showing the directional migration of the magnetic-responsive dual-layered jellyfish microparticles under a strong magnetic field. The scale bars are 1000 μm.

To impart the particles with stimuli-triggered motion ability, functional GO/NIPAM composite hydrogels were employed as the component of the dual-layered particles so that they could respond to NIR. Benefiting from the photothermal effect of the GO/NIPAM hydrogel, the particles could undergo contraction under NIR irradiation. It was found that when the particle was exposed to the NIR light, the bioinpired jellyfish appeared to shrink within about 2 s, and it would return to the swelling state within about 1 s. As a result, the particles could be propelled to move forward. We validated this process by tracking the displacement of a single particle under NIR irradiation, as shown in Fig. 5A and B and Movie S3. Moreover, by incorporating Fe_3_O_4_ nanoparticles into the innermost layer, the particles could be endowed with additional magneto-responsiveness. As the Fe_3_O_4_ nanoparticles were dispersed in the inner layer of the particles and occupied a large proportion in the head, the response of the particles to the magnetic field should be asymmetric, thus facilitating controllable movement. When the particles were placed in a relatively weak field (the magnet was set 4 cm from the jellyfish), they reorientated and turned their heads toward the magnet (Fig. 5C and D and Movie S4). When the magnetic field was further enhanced (moving toward the particles to 2 cm), the particles would move directionally to the magnet side, as shown in Fig. 5E and F and Movie S5. In general, with the increase of the concentration of Fe_3_O_4_ nanoparticles, the average velocity of the jellyfish increased accordingly (Fig. S7). These features demonstrated the prominent controllability of the bioinspired jellyfish-like microparticles under multiple external stimuli.

**Fig. 6.**
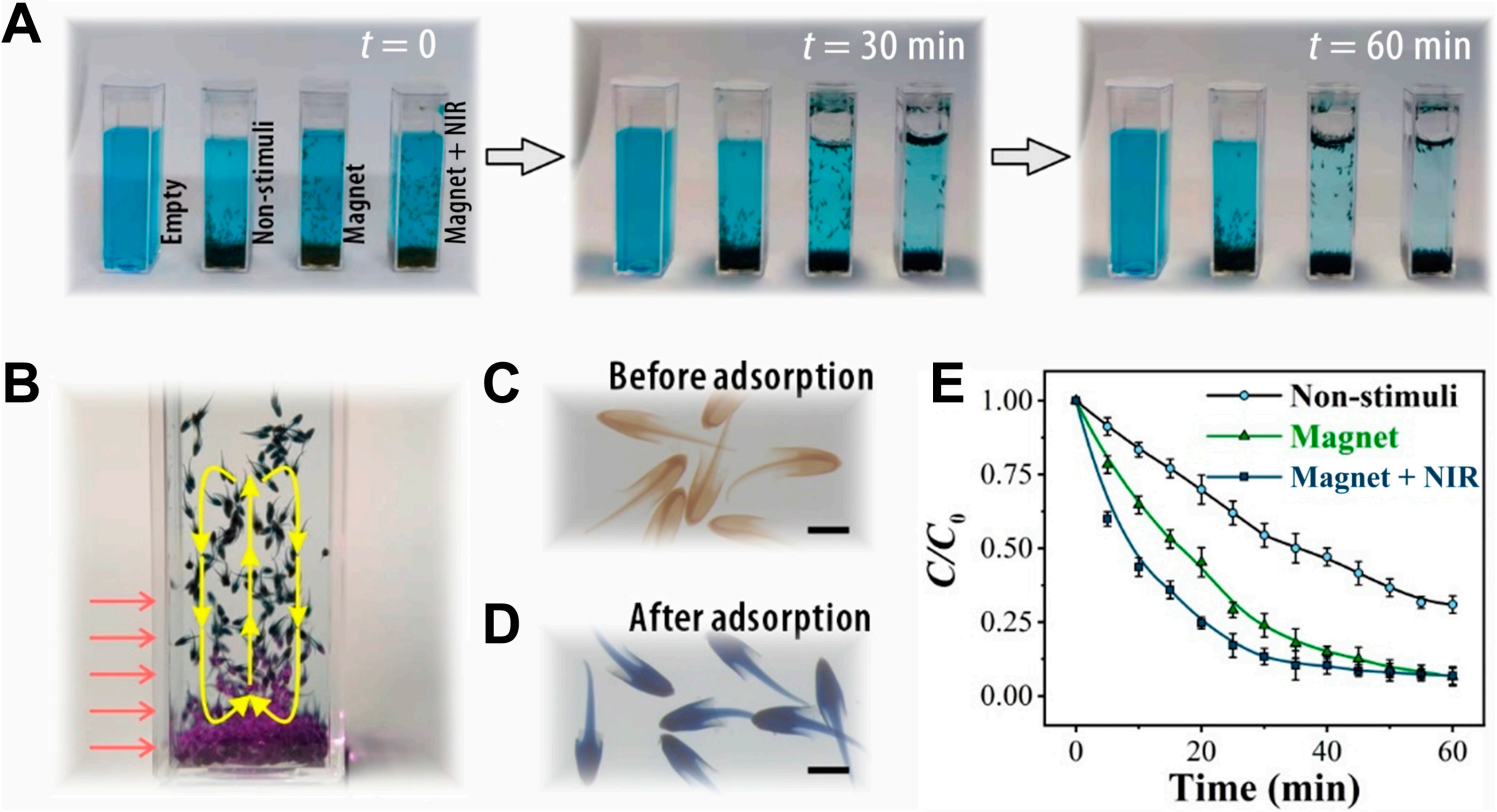
Adsorption processes of the dual-layered jellyfish particles under different conditions. (A) The adsorption process of MB using magnetic GO/NIPAM dual-layered jellyfish particles under different stimulation modes. (B) The circulation of a swarm of particles under NIR stimulation. (C and D) Microscopic images of the particles (C) before and (D) after MB adsorption. (E) The MB adsorption kinetics of the dual-layered jellyfish particles. *C* was the real-time concentration, and *C*_0_ was the initial concentration of MB. The scale bars are 1000 μm.

Because of the versatile motion controllability features, the multi-stimuli-responsive jellyfish particles were expected to be applied in the adsorption of water impurities. Furthermore, because GO owns extraordinarily large surface areas and large numbers of polar groups, it has a natural advantage in impurity adsorption [44,45]. It was found that with the absence of external stimuli, the jellyfish particles showed faster adsorption than spherical particles with the same volume. This could be attributed to the larger surface area of the former (Fig. S8). We then tested the adsorption process using these microparticles with movement under combinational control of magnetic field and NIR (Fig. 6). The particles could be drawn from one side of the cuvette to the other by alternating the direction of the magnet (Fig. S9 online). Moreover, NIR irradiation could be exerted to cause a circulation of the swarm of particles, which was probably due to the cooperation of the particle movement and Rayleigh–Benard effect. This thus enhanced the contact of the particles with the dyes in the solution (Fig. 6B). Benefiting from these effects, the particles under dual stimuli achieved the best adsorption performance, as manifested by the color change (Fig. 6A). It was also found that with sufficient adsorption, the color of the particles changed from brown to blue, indicating that the pollutants were adsorbed by these particles, as shown in Fig. 6C and D. Then, we studied the adsorption kinetics of these particles with different stimulation modes using a model pollutant methylene blue (MB). We measured the real-time concentration of MB (*C*) relative to its initial concentration (*C*_0_) at different time points for each group, as plotted in Fig. 6E. It was found that the magnetic stimuli group showed faster and more effective adsorption capability than the nonstimuli group due to efficient movement. In addition, the magnet/NIR stimuli group showed superior adsorption capability even to the magnetic stimuli group, which suggested that the particles under combinational control could have a larger chance of contact with the pollutants in the solution.

## Discussion

In summary, we have developed novel bioinspired jellyfish microparticles from piezoelectric microfluidics. The jellyfish microparticles were derived from specific flow configurations through the piezoelectric pulsation of a fluid jet. The size and morphology of the particles were readily overmastered by altering the piezoelectric and microfluidic parameters. Furthermore, multi-compartment jellyfish microparticles with dual-layer structures were facilely achieved by tailoring the channel geometry of the microfluidics. Moreover, by employing the GO/NIPAM hydrogel precursor and magnetic nanoparticles into the flow templates, the resultant jellyfish microparticles were imparted with stimuli-responsive motion ability. Such distinct characteristics of the bioinspired particles have demonstrated their superiority in water purification and have been promising materials for other related areas including biomimetics, smart robotics, etc. Hence, future endeavors can be made in the systematic investigation of the locomotion of these jellyfish particles with variable morphologies and stimuli conditions to achieve more efficient adsorption efficiency. In addition, other materials and control methods can also be developed to realize intelligent drug delivery in, e.g., constrained blood vessels. The potential applications of this strategy can be expanded by developing alternative stimuli-responsive materials (e.g., liquid crystal) and designing more complex network topologies. We believe that this study would also shed light on the microfluidic fabrication of anisotropic microparticles that would open an avenue to a wide range of applications.

## Materials and Methods

### Materials

PEGDA (average *M*_n_ = 700), *N*,*N*′-methylenebis (acrylamide) (BIS), and photoinitiator 2-hydroxy-2-methylphenylpropanone (HMPP) were all procured from Sigma-Aldrich. Fluorescent polystyrene (PS) nanoparticles (F8805 and F8811) were procured from Invitrogen. GO solution (2 mg/ml) was bought from XF NANO Co. Ltd. NIPAM (97%) and methylene blue (MB) were bought from Macklin. Photoinitiator lithium phenyl(2,4,6-trimethylbenzoyl) phosphinate (LAP) was derived from Aladdin. Magnetic nanoparticles were synthesized by a hydrothermal method. Silicone oil (50 cSt) was purchased from Shin-Etsu Chemical Co. Ltd.

### Piezoelectric microfluidics construction

The microfluidics was constructed by coaxially aligning 2 capillaries on a glass slide. The inner circular capillary (capillary 2), with an inner and outer diameter of 580 μm and 1.0 mm, respectively (World Precision Instruments), was tapered by a capillary puller (Sutter Instrument, P-97) and then sanded to the desired diameter (~200 μm) using a microforge (Narishige, MF-830). The outer capillary (capillary 3) was also cylindrical (inner diameter: 1560 μm, outer diameter: 2 mm). Capillary 2 served as the injection tube, and capillary 3 was used as the collection tube. For the generation of dual-layered particles, another capillary (capillary 1) with an orifice diameter of about 150 μm was coaxially inserted into capillary 2, and these 2 capillaries were coaxially aligned in capillary 3 to form a 2-stage nested configuration. Capillary 1 and capillary 2 served as the innermost and middle injection channels, respectively, and capillary 3 served as the outer fluid channel. The joints of these capillaries were sealed by transparent epoxy resin where necessary. To generate jellyfish ligament templates, a piezoelectric stack (PSt150/7/20VS12, CoreMorrow) was employed to transmit the sinusoidal signal to the corresponding fluid by a thin PTFE film (the thickness is 0.1 mm), as illustrated in Fig. S10. The piezoelectric stack was applied to the microfluidic chip by modulating the flexible tube to the injection tube with the syringe. The piezoelectric stack actuator was connected to a signal generator (Siglent, SDG 2000X) via a power amplifier (CoreMorrow, E-05) to program the amplitudes and frequencies. In this paper, the magnification of the power amplifier was fixed as 12×, and the voltages mentioned throughout the manuscript were in the form of *V*_p–p_ (peak-to-peak) of the signal generator.

### Jellyfish particle generation

For the generation of single-layer jellyfish particles, the inner phase was an aqueous solution of PEGDA (15% v/v) with photoinitiator HMPP (1% v/v) and the outer phase was deionized water. Both solutions were driven into the corresponding channels by 2 syringe pumps (Longer, LSP01-2A), and the inner phase was connected to the piezoelectric stack actuator. For observing the dynamics of the generation of the ligament template, the flow rates were *Q_i_* = 5 ml/h and *Q_o_* = 65 ml/h. For the generation of simple jellyfish particles, the typical set of flow rates was *Q_i_* = 5 ml/h and *Q_o_* = 55 ml/h. For the generation of the dual-layered jellyfish particles, the innermost phase and the middle phase were both aqueous solutions of PEGDA (15% v/v) with HMPP (1% v/v), and the outer phase was deionized water. Red and green fluorescent PS nanoparticles were added to the innermost and middle phases, respectively (each with a concentration of 0.5% v/v), for fluorescent imaging. These solutions were all pumped to the corresponding channels, and the middle phase was actuated by the piezoelectric stack actuator. A typical set of the flow rates was *Q_i_* = 1.5 ml/h, *Q_m_* = 3.5 ml/h, and *Q_o_* = 55 ml/h. For the generation of multi-stimuli-responsive particles, the innermost phase was a mixture solution of GO (1 mg/ml), NIPAM (4% v/v), PEGDA (8% v/v), BIS (corresponding to 1/30 to the mass of NIPAM), and LAP (0.1% v/v); the middle phase was a mixture solution of GO (2 mg/ml), NIPAM (8% v/v), PEGDA (8% v/v), BIS (corresponding to 1/30 to the mass of NIPAM), and LAP (0.1% v/v); and the outer phase was deionized water. The flow rates were *Q_i_* = 1.5 ml/h, *Q_m_* = 3.5 ml/h, and *Q_o_* = 55 ml/h. In all cases, the jellyfish-like ligament templates were rapidly solidified downstream upon in situ UV irradiation with double heads (EXFO OmniCure Series 1000, 365 nm, 100 W).

### Spherical particle generation

For spherical particle generation, we employed the same microfluidics setup used for the generation of single-layered jellyfish particles. The inner phase was a mixture solution of GO (2 mg/ml), NIPAM (8% v/v), PEGDA (8% v/v), BIS (corresponding to 1/30 to the mass of NIPAM), and LAP (0.1% v/v). The outer phase was silicon oil (50 cSt). To compare its adsorption efficiency with jellyfish particles, the volume of the spherical and jellyfish-like particles was adjusted to be consistent. Thus, the diameter of the spherical particle was calculated to be about 780 μm under a condition for preparing jellyfish particles (*Q_i_* = 5 ml/h, *f* = 6 Hz). In this experiment, the inner and outer flow rates were set as *Q_i_* = 4 ml/h and *Q_o_* = 80 ml/h, respectively. After collection, the resultant particles were washed 10 times to remove the silicone oil for further use.

### Characterization

The jellyfish template generation dynamics was recorded by a microscope equipped with a fast camera (Acuteye). The optical and fluorescence microscopic images of the bioinspired particles were captured using a fluorescent microscope (OLYMPUS, CKX53) with a charge-coupled device. Cross-sectional fluorescence images of the particles were taken using a laser scanning confocal microscope (Leica, STELLARIS). The microstructures of the solidified jellyfish microparticle were captured by SEM (HITACHI, SU8010).

### Studying the adsorption kinetics of the bioinspired particles

MB was used as the model pollutant to test the adsorption performance of the resultant bioinspired particles. The adsorption experiments were performed at room temperature (25 °C). The MB concentration in water was obtained at 662 nm using a microplate reader (Epoch, BioTek). For the comparison experiment for sphere and jellyfish, we collected 2/3 ml of adsorbents in each group and then plated them in petri dishes; the initial MB concentration was 5 mg/l, and the volume was 12 ml. For the comparison experiment for different stimulations, we collected 0.5 ml of adsorbents in each group and then put them in the centrifuge tube; the initial MB concentration was 10 mg/l, and the volume was 7 ml. The number of replicates was 3. For the magnetic stimuli, the direction of the magnetic field (4000 GS; 4 cm × 2 cm × 0.5 cm, distance of 0 cm between the container) was alternated every 5 min to pull the jellyfish microparticles from one side of the container to the other side. For NIR stimuli, the particles were exposed to laser (808 nm) irradiation for 1 min every 5 min at a distance of 10 cm after changing the magnet direction. For dual stimuli, it is a combination of the above 2 stimuli methods.

## Data Availability

All data used to support the findings in the paper and the Supplementary Materials are available from the corresponding authors upon reasonable request.

## Supplementary Materials

Supplementary 1 Fig. S1. (A) Digital photograph of the microfluidic device and (B) the microscopic image showing the coaxial channel configuration. The scale bar is 1000 μm. Fig. S2. Schematic illustration of the piezoelectric microfluidic platform and jellyfish particles’ generation process. Fig. S3. (A) High-speed real-time images of the dynamic behaviors of the jellyfish ligament template under different inner flow rates; (B) plot of the width of the jellyfish-like ligament template as a function of the inner flow rate. The scale bar is 500 μm. Fig. S4. Schematic illustration of the different UV irradiation positions and the resultant jellyfish particles with different morphologies. The scale bars are 1000 μm in the left panel and 500 μm in the right panel. Fig. S5. (A) Digital photograph of the microfluidic device for the generation of dual-layered jellyfish particles and (B) the microscopic image showing the coaxial channel configuration. The scale bar is 1000 μm. Fig. S6. Composition control of the jellyfish particles by tuning the ratio of the innermost and middle flow rates. *Q_i_* + *Q_m_* = 5 ml/h, *Q_o_* = 56 ml/h. The scale bar is 1000 μm. Fig. S7. The relationship between the jellyfish average velocity (moving 2.4 cm) and the concentration of Fe_3_O_4_ under the same magnetic field conditions. Fig. S8. (A) Optical microscopic images of the spherical and jellyfish-like particles with the same volume; (B) the absorption kinetic of spherical and jellyfish-like particles in petri dishes without stimulation. The scale bar is 1000 μm. Fig. S9. The particles could be pulled between 2 sides by alternating the direction of the magnet. Fig. S10. The schematic of the configuration of piezoelectric vibration. The piezoelectric vibration can be coupled into the fluid flow through a thin film. When ∆*Q* > *Q_i_*, it can produce the flow reversion. Fig. S11. (A) Three typical flow regimes and (B) phase diagram of the different flow regimes as a function of the piezoelectric amplitude and frequency *f*. Fig. S12. The schematic of the evolution process of the jellyfish-like ligament template with Poiseuille advection. Note S1. Flow mechanism of the jellyfish-like liquid configuration.
