# Supplementary material for: Bioinspired Jellyfish Microparticles from Microfluidics
Source: Research (Wash D C). 2023 Jan 16;6:0034. doi: 10.34133/research.0034 (PMC10076059; doi:10.34133/research.0034)
Supplement: Supplementary 1 — Fig. S1. (A) Digital photograph of the microfluidic device and (B) the microscopic image showing the coaxial channel configuration. The scale bar is 1000 μm. Fig. S2. Schematic illustration of the piezoelectric microfluidic platform and jellyfish particles’ generation process. Fig. S3. (A) High-speed real-time images of the dynamic behaviors of the jellyfish ligament template under different inner flow rates; (B) plot of the width of the jellyfish-like ligament template as a function of the inner flow rate. The scale bar is 500 μm. Fig. S4. Schematic illustration of the different UV irradiation positions and the resultant jellyfish particles with different morphologies. The scale bars are 1000 μm in the left panel and 500 μm in the right panel. Fig. S5. (A) Digital photograph of the microfluidic device for the generation of dual-layered jellyfish particles and (B) the microscopic image showing the coaxial channel configuration. The scale bar is 1000 μm. Fig. S6. Composition control of the jellyfish particles by tuning the ratio of the innermost and middle flow rates. Qi + Qm = 5 ml/h, Qo = 56 ml/h. The scale bar is 1000 μm. Fig. S7. The relationship between the jellyfish average velocity (moving 2.4 cm) and the concentration of Fe3O4 under the same magnetic field conditions. Fig. S8. (A) Optical microscopic images of the spherical and jellyfish-like particles with the same volume; (B) the absorption kinetic of spherical and jellyfish-like particles in petri dishes without stimulation. The scale bar is 1000 μm. Fig. S9. The particles could be pulled between 2 sides by alternating the direction of the magnet. Fig. S10. The schematic of the configuration of piezoelectric vibration. The piezoelectric vibration can be coupled into the fluid flow through a thin film. When ∆Q > Qi, it can produce the flow reversion. Fig. S11. (A) Three typical flow regimes and (B) phase diagram of the different flow regimes as a function of the piezoelectric amplitude and frequency f. Fig. [file research.0034.f1.docx]

Supplementary Materials


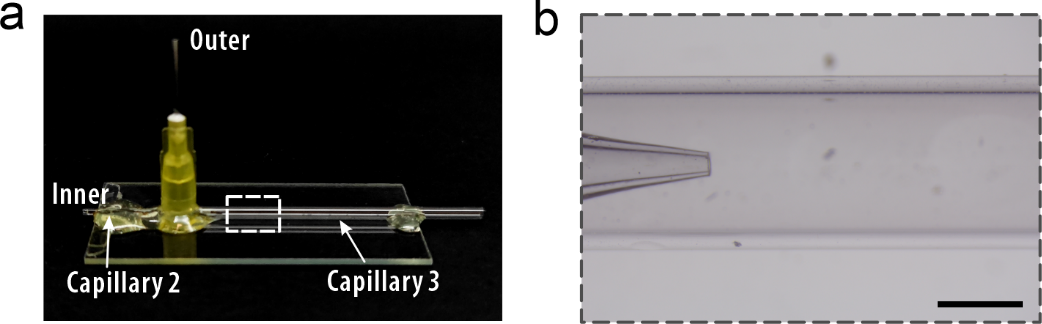


**Figure S1.** (a) Digital photograph of the microfluidic device and (b) the microscopic image showing the coaxial channel configuration. The scale bar is 1000 *µ*m.


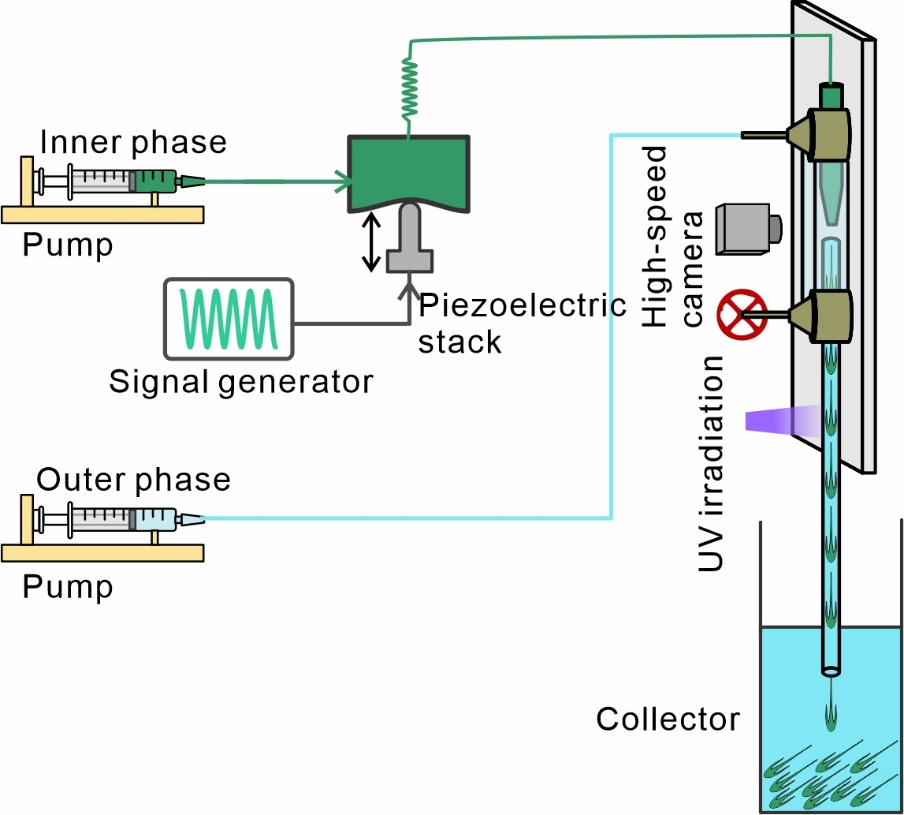


**Figure S2.** Schematic illustration of the piezoelectric microfluidic platform and jellyfish particles’ generation process.


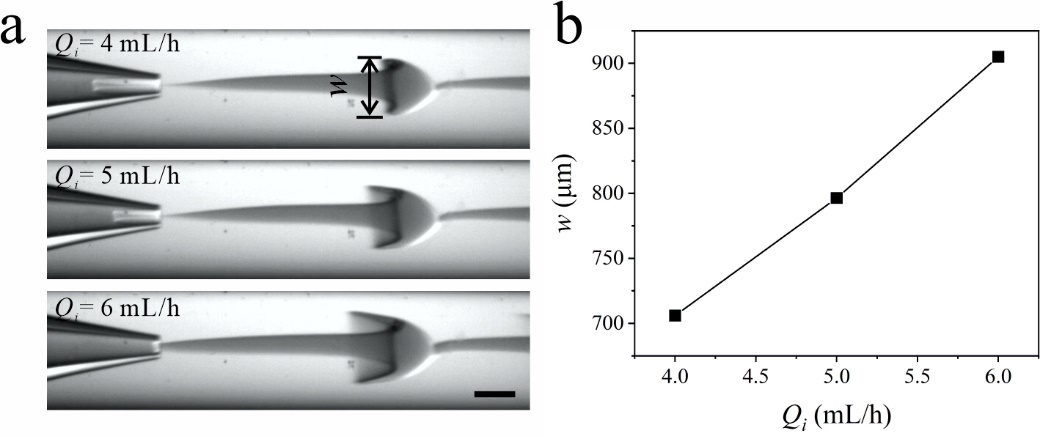


**Figure S3.** (a) High-speed real-time images of the dynamic behaviors of the jellyfish ligament template under different inner flow rates; (b) plot of the width of the jellyfish-like ligament template as a function of the inner flow rate. The scale bar is 500 *µ*m.


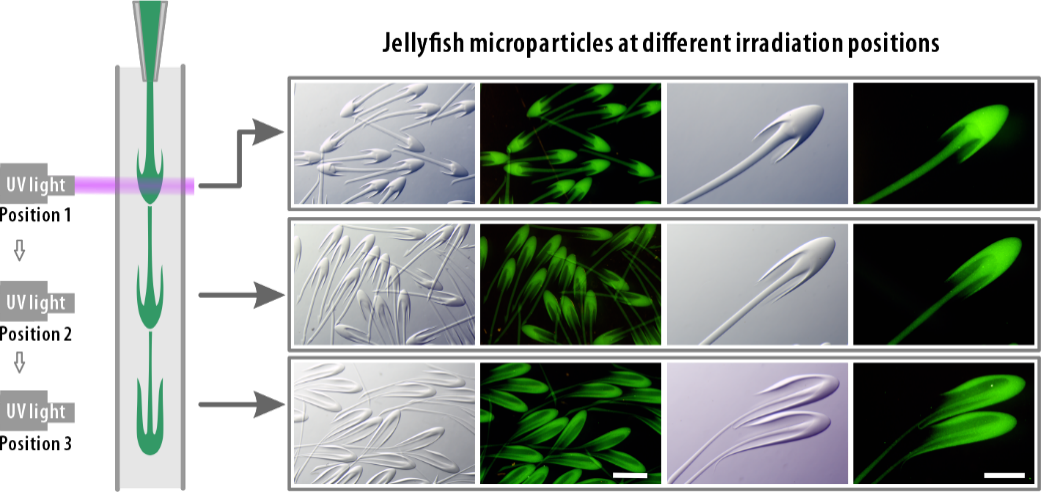


**Figure S4.** Schematic illustration of the different UV irradiation positions and the resultant jellyfish particles with different morphologies. The scale bars are 1000 *µ*m in the left panel and 500 *µ*m in the right panel.


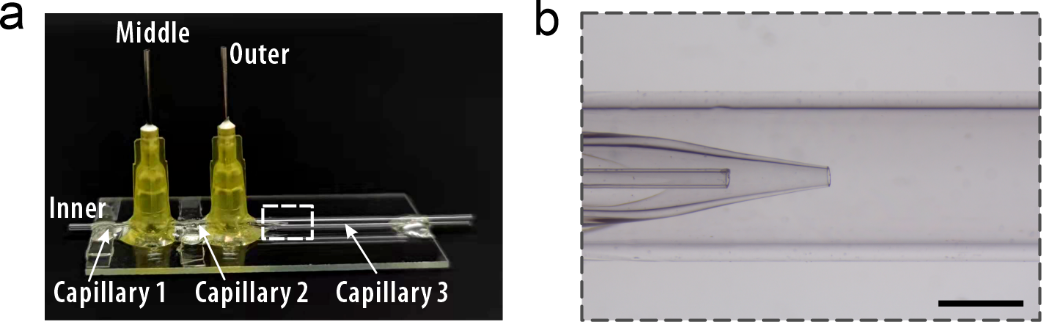


**Figure S5.** (a) Digital photograph of the microfluidic device for the generation of dual-layered jellyfish particles and (b) the microscopic image showing the coaxial channel configuration. The scale bar is 1000 *µ*m.


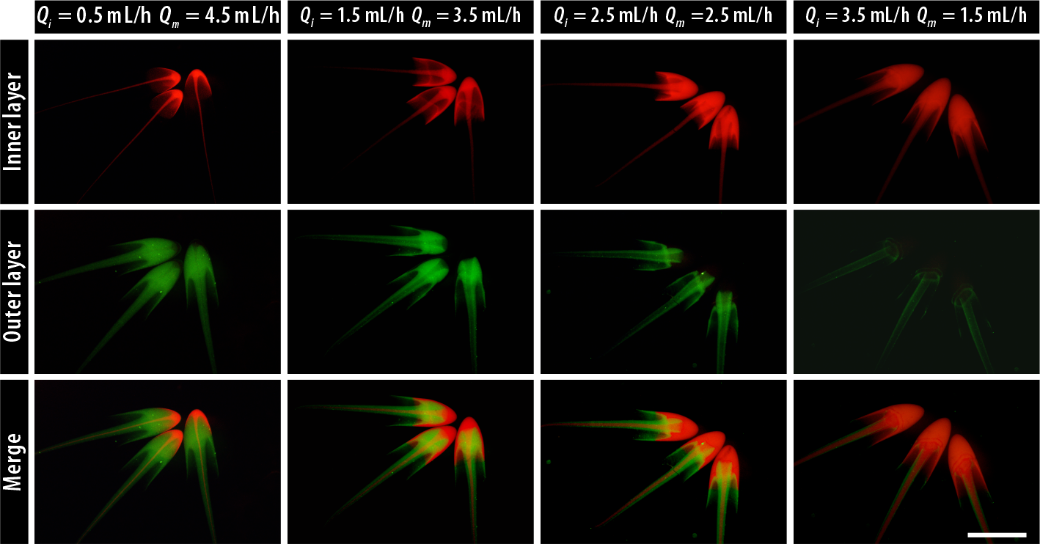


**Figure S6.** Composition control of the jellyfish particles by tuning the ratio of the innermost and middle flow rates. *Q_i_*+*Q_m_* = 5 mL/h, *Q_o_* = 56 mL/h. Scale bar is 1000 *µ*m.


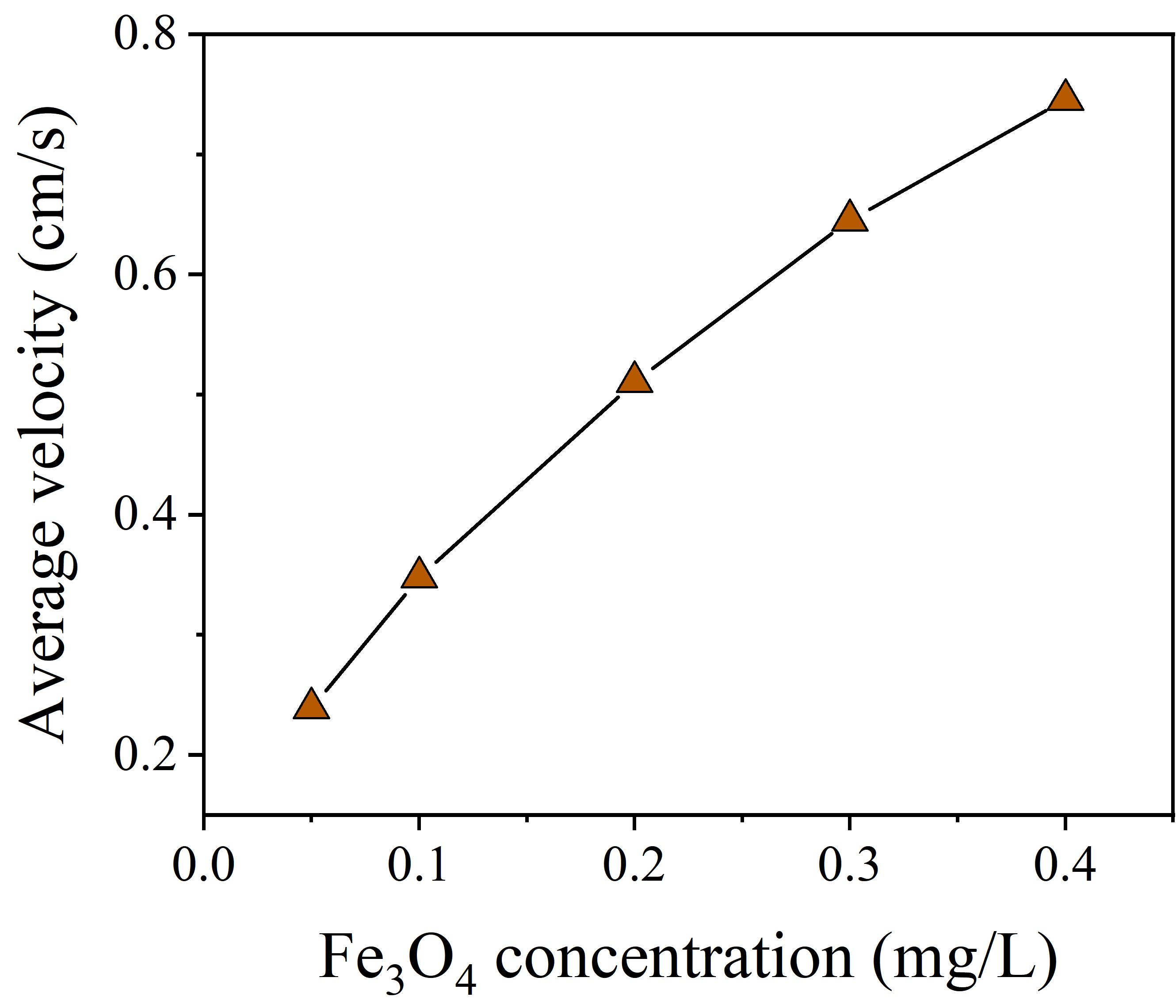


**Figure S7.** The relationship between the jellyfish average velocity (moving 2.4 cm) and the concentration of Fe_3_O_4_ under the same magnetic field conditions.


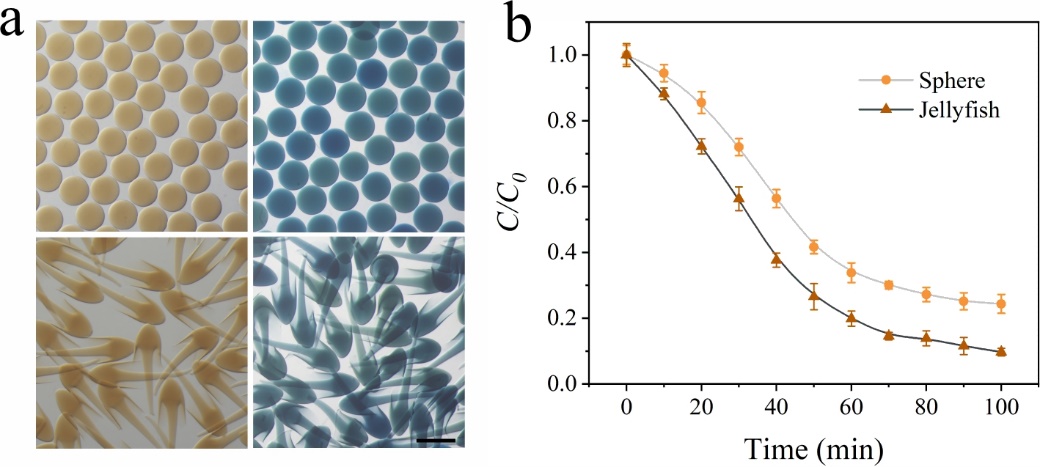


**Figure S8.** (a) Optical microscopic images of the spherical and jellyfish-like particles with the same volume; (b) the absorption kinetic of spherical and jellyfish-like particles in petri dishes without stimulation. The scale bar is 1000 *µ*m.


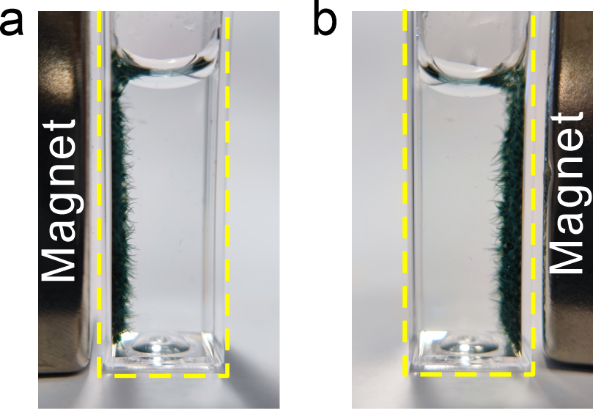


**Figure S9.** The particles could be pulled between two sides (from the left to the right) by alternating the direction of the magnet.

**Note S1. Flow mechanism of the jellyfish-like liquid configuration**

The generation process of single-layered jellyfish liquid configuration was studied as a model. An inner aqueous pre-gel solution (15% PEGDA, *η* = 1.85 mPa.s, *ρ* = 1.023 g/cm^3^) and the outer fluid of deionized water (*η* = 0.99 mPa.s, *ρ* = 0.998 g/cm^3^) were simultaneously driven to the corresponding channels, respectively, and flowed in the same direction to form a steady jet downstream in the collection capillary. For the typical flow condition, the Reynolds number ${(Re}_{\text{i}}=\frac{\rho_{\text{i}} u_{\text{i}} L}{\eta_{\text{i}}})$is 4.89. Upon piezoelectric vibration, the flow rate of inner phase was modulated consistently with the oscillation pulsations **Figure S10**. The inner flow after piezoelectric vibration regulation can be expressed as follows:

$Q_{i}(t)=Q_{i}+\Delta Q(t)$,

where *Q_i_* is the constant inner flow rate from the pump, $\Delta Q(t)$ is the disturbance amplitude of the flow from the piezoelectric vibration, and *f* is the frequency of flow fluctuations. The film was squeezed and deformed by the piezoelectric stack, which can be regarded as the volume change of a cone:

$V=\frac{1}{3}{\pi r}^{2}[h+Asin\left( 2\pi ft \right)]$,

where *r* represents the radius of the film, *h* is initial height of the film, and *A* is the displacement of the piezoelectric actuator. Thus, the volume change can be estimated as

$\Delta V=\frac{1}{3}{\pi r}^{2}Asin\left( 2\pi ft \right)$.

Since the displacement of the piezoelectric actuator is proportional to the input voltage *U* (that is, *A = kU,* where *k* is a constant determined by the properties of piezoelectric ceramics) and is of small value. The flow rate disturbance can be deduced as

$\Delta Q(t)=\frac{d\Delta V}{dt}=\frac{2}{3}{\pi^{2}r}^{2}kUfcos\left( 2\pi ft \right)$.

Thus, the flow disturbance can be regarded as the approximately linear relationship with the applied voltage.


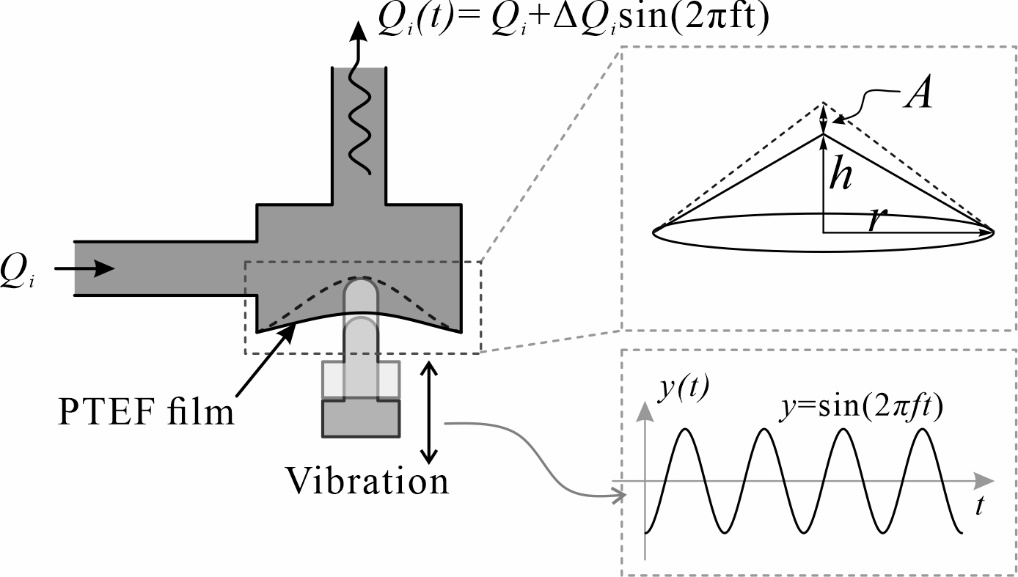


**Figure S10**. The schematic of the configuration of piezoelectric vibration. The piezoelectric vibration can be coupled into the fluid flow through a thin film. When △*Q*>*Q_i_,* it can produce the flow reversion.

It was found that by changing the piezoelectric parameters (frequency and amplitude), the resultant templates could form three typical flow regimes, including the straight-jet regime, wavy-jet regime, and jellyfish-like regime. The fluid dynamic regimes were summarized as a function of the piezoelectric frequency and amplitude, as shown in **Figure S11**. Specifically, in the absence of vibrations, and with typical flow rates of *Q_i_* = 5 mL/h and *Q_o_* = 55 mL/h, a stable jet would form downstream. As we introduce piezoelectric vibrations with low amplitude, at frequencies of *f* = 6 Hz, wavy jets took place with shape instabilities because of inner fluid oscillating (the jet does not break up into separate ligaments).


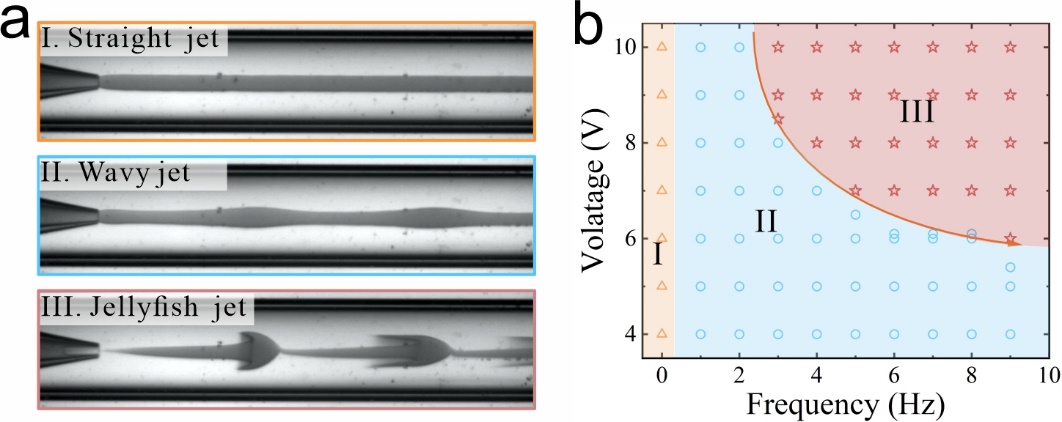


**Figure S11**. (a) Three typical flow regimes and (b) phase diagram of the different flow regimes as a function of the piezoelectric amplitude and frequency *f*.

However, when the vibration amplitude was large enough, the fluctuation caused the inner jet to periodically retract and go forward, and then the jet would break up into dispersed ligaments with a varicose head and thin-thread tail and subsequently deformed into a jellyfish-like shape in accordance with Poiseuille advection, as shown in **Figure S12**.


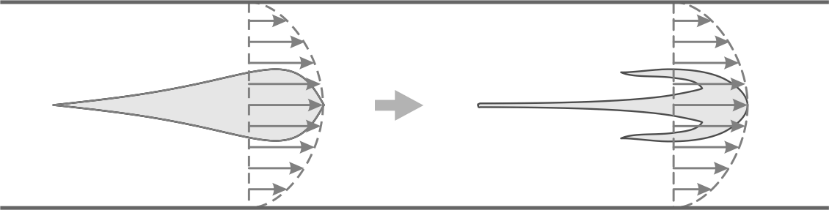


**Figure S12.** The schematic of the evolution process of the jellyfish-like ligament template with Poiseuille advection.

In the straight-jet regime, the diameter of the jet and the flow rates of the fluids satisfy the following relation^[1]^:

$d=D\left[ 1-\left( \frac{\text{Q}_{\text{o }}}{\text{Q}_{\text{i }}+\text{Q}_{\text{o }}} \right)^{1/2} \right]^{1/2}$ (1)

where *Q_i_* and *Q_o_* are the volumetric flow rates of the inner and outer phase, respectively, and *D* is the inner diameter of the collection tube. Since the frequency of the jelleyfish-like ligament is directly related to the piezoelectric frequency *f*, the length of the ligament *l* can be writen as:

$l=\frac{4Q_{i}}{\pi d^{2}f}$ (2)

1. A. Sauret, H. C. Shum, InterInt. J. Nonlinear Sci. Numer. Simul. 2012, 13.
